# The impact of the size of the energy deficit on the rate of body weight in 6 months and willingness to continue reduction program conducted online–An intervention study

**DOI:** 10.1002/fsn3.4442

**Published:** 2024-09-12

**Authors:** Jakub Woźniak, Katarzyna Woźniak, Kornelia Pajtel, Michał Wrzosek, Dariusz Włodarek

**Affiliations:** ^1^ Department of Dietetics, Institute of Human Nutrition Sciences Warsaw University of Life Sciences (WULS–SGGW) Warsaw Poland; ^2^ Centrum Respo Warsaw Poland

**Keywords:** diet intervention, energy deficit, human, lose weight, obesity, online intervention, overweight, physical activity

## Abstract

Overweight and obesity are among the most serious public health problems, making new methods for their prevention, as well as treatment, constantly being sought. This study was designed as a 6 month intervention study. The main objective was to evaluate the effect of an energy deficit (10%, 20%, and 25%—groups D10, D20, and D25, respectively) on the rate of weight loss and waist and hip circumferences. The protocol was completed by 180 participants. Men as well as women comprised 90 patients each. The mean body mass index (BMI) was 30.5 ± 5.0 kg/m^2^ (min 21.3–max 49.2). Among the patients, there were 86 with a BMI indicating overweight. Fifty‐four patients had class 1 obesity, while the remaining 30 patients had class 2 or greater obesity. After 6 months of intervention, the D10 group noted a 7.6% (median) reduction in weight, the D20 group a 9.9% (median) reduction in weight, and the D25 group a 10.3% (median) reduction in weight. After the intervention, 51.7% of patients chose to continue further weight reduction already outside the research protocol. Key factors influencing the willingness to stay on the diet longer than 6 months were higher baseline body weight and/or higher BMI and a rate of weight loss of at least 1.5% of body weight per month. In summary, it appears that a larger energy deficit (on the order of 20%–25%) is most appropriate in terms of weight loss lasting 6 months and motivation for continued therapy.

## Graphical abstract

Key factors influencing the willingness to stay on the diet longer than 6 months were higher baseline body weight and/or higher body mass index (BMI) and a rate of weight loss of at least 1.5% of body weight per month. In summary, it appears that a larger energy deficit (on the order of 20%–25%) is most appropriate in terms of weight loss lasting 6 months and motivation for continued therapy.

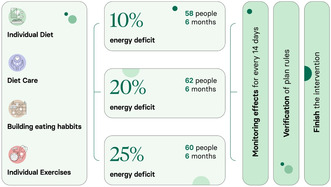

## 1 INTRODUCTION

Excessive weight and obesity represent prominent global public health challenges, prompting continuous endeavors for the development of novel preventative and therapeutic modalities (World Health Organization, 2020). One of the most common therapeutic interventions is the development of nutritional intervention strategies to reduce excess body weight and improve health outcomes (Kim, 2021). The lack of full effectiveness in following the recommendations of specialists and the constantly worsening statistics on overweight and obesity mean that new forms of contact with patients and ways to support them in weight reduction therapy are being sought. A good space for such activities may turn out to be the online environment (Kręgielska‐Narożna et al., 2013). Using the Internet, a range of health behavior services can be provided. The global count of Internet users surpasses 4 billion patients, and as a result, the activity of dietitians in the online world offers the opportunity to reach many more people than traditional solutions (Kamiński et al., 2020). Recent reports indicate that interventions, aimed at improving health in general (including nutrition), carried out via the Internet yield the expected results. Moreover, personalized feedback provided by a dietitian in an online fashion increases patient engagement in the therapeutic process and is associated with a greater chance of significant reductions in excess body weight. Long‐term therapeutic intervention conducted online with patients with overweight and obesity appears to be an effective way to lose weight and improve health parameters (Beleigoli et al., 2020; Brug et al., 2005; Tzelepis et al., 2021).

The main way to influence the reduction of excessive body weight when working with patients with obesity is to reduce the energy value of the diet so as to achieve a negative energy balance.

The size of the energy deficit and its individual adjustment for the patient are undeniably the most important factors affecting the effectiveness of the therapeutic process (Kim, 2021). Guidelines of experts from various countries on the treatment of obesity recommend gradual weight reduction to limit the risk of returning to baseline weight later. They point out that too much restriction in the energy value of the diet, which allows faster weight loss, can increase the risk of deviation from the diet and weight gain again (yo‐yo effect) (National Clinical Guideline Centre (UK), 2014; Durrer Schutz et al., 2019; Bąk‐Sosnowska et al., 2022). In accordance with the European clinical practice guidelines concerning the management of adult obesity (Durrer Schutz et al., 2019), weight loss should be achievable, individualized, and long‐term. In addition, the recommended magnitude of weight reduction has been reported to be 5%–15% over a period of 6 months. The latest position of the Polish Society for the Treatment of Obesity indicates that the optimal goal is to reduce body weight at a rate of 1 kg in the first week and 0.5 kg/week in subsequent weeks, with an energy deficit of 500–600 kcal, and the energy value of the recommended diet must not be less than that necessary to maintain basal metabolic rate (BMR) (Bąk‐Sosnowska et al., 2022). Reducing excess body weight has many beneficial health effects, but it is still unclear whether they depend on its rate. Current studies (Fogarasi et al., 2022; Vink et al., 2016) shed a different light on the current recommendations, indicating that the rate of weight loss did not affect the return to initial body weight at a later time. Moreover, losing the same amount of weight at different rates did not significantly affect changes in body composition and metabolism, and small differences are unlikely to be clinically relevant to the long‐term treatment of obesity. However, the use of very low‐calorie diets (VLCDs) may result in a greater percentage loss of lean body mass, as opposed to diet plans that assume a smaller energy deficit (Vink et al., 2016). Strategies for weight loss and maintenance should certainly be individualized, and it is up to dietitians to choose the best approach based on patient preferences (Kim, 2021). Taking into account the above facts, we formulated the following hypothesis: an energy deficit of 20% is the most effective in terms of the rate of weight loss in a 6 months intervention aimed at weight loss. The aim of this study was to assess the impact of varying energy deficits (by 10%, 20%, or 25%) on the rate of weight loss and anthropometric parameters (waist and hip circumferences). An additional objective of the study was to evaluate the effectiveness of collaboration with a dietitian using only online communication on the effectiveness of weight reduction. In addition, we assessed the influence of factors, such as age, body mass index (BMI) before as well as after the intervention, the degree of weight reduction, waist and hip circumferences before as well as after the intervention, and weight reduction in the last month of the intervention on the patient's willingness to continue working with a dietitian aimed at further weight reduction.

## 2 METHODS

### 2.1 Study design

Our study is a 6 months intervention involving humans. The data collection lasted from July 2022 to January 2023. This is a randomized intervention trial conducted 100% online. The intervention was carried out in three groups in which energy deficit and regular physical activity were used for 6 months under the constant supervision of a dietitian, conducted online. The energy deficit was set at three levels: 10%, 20%, and 25% to individual energy needs in order to determine the effectiveness of reducing excess body weight in an online intervention at various levels. Our primary focus revolved around evaluating fluctuations in weight throughout the duration of the program through relative metrics, specifically the percentage reduction in baseline body weight, rather than absolute measures denoted in kilograms. Utilizing this approach facilitates a more objective analysis of the phenomenon of weight loss. The study was approved by the local Ethics and Scientific Research on Humans Commission of Faculty of Human Nutrition and Consumer Sciences—SGGW (Warsaw University of Life Sciences) approved the research project (approval number: 35/2021). All subjects signed consent form to participate in the study.

### 2.2 Sample

People participating in the study were recruited from among people attending an online clinic dealing with dietary therapy of obesity. Inclusion criteria were: ages of 18 and 50 years, excessive body weight (BMI >25), no weight loss in the last 24 months, no muscle injuries, access to a computer and/or telephone, no contraindications from a doctor or physiotherapist to engage in physical activity, willingness to reduce body weight, readiness to modify the diet, undertake regular physical exercise, have a bathroom scale with an appropriate certificate (due to the measurements being performed by the participating people themselves), and readiness to participate in the study before 6 months. The surveyed people volunteered to participate in the study. A total of 224 people signed up, but during the inclusion in the study and presentation of its detailed assumptions, 44 people withdrew without giving any reason. Recruitment was completed when 180 people (90 women and 90 men) were included in the study. It was assumed that the number of people in each of the three groups of the intervention study should be approximately 60 people, with an equal proportion of women and men in each of them. Groups of this size allow for statistical analysis.

### 2.3 Study procedure and intervention characteristics

The source of data was a nutritional form completed by 180 patients included in the intervention study. Body weight and body circumference measurements were performed at the beginning and during the intervention by patients themselves. The intervention (RESPO method) consisted of an exercise program and diet with a 10%, 20%, or 25% energy deficit group D10, D20, and D25, respectively. Study participants, after enrollment in the study, were randomly assigned to the respective groups, separately men and women.

**FIGURE 1:**
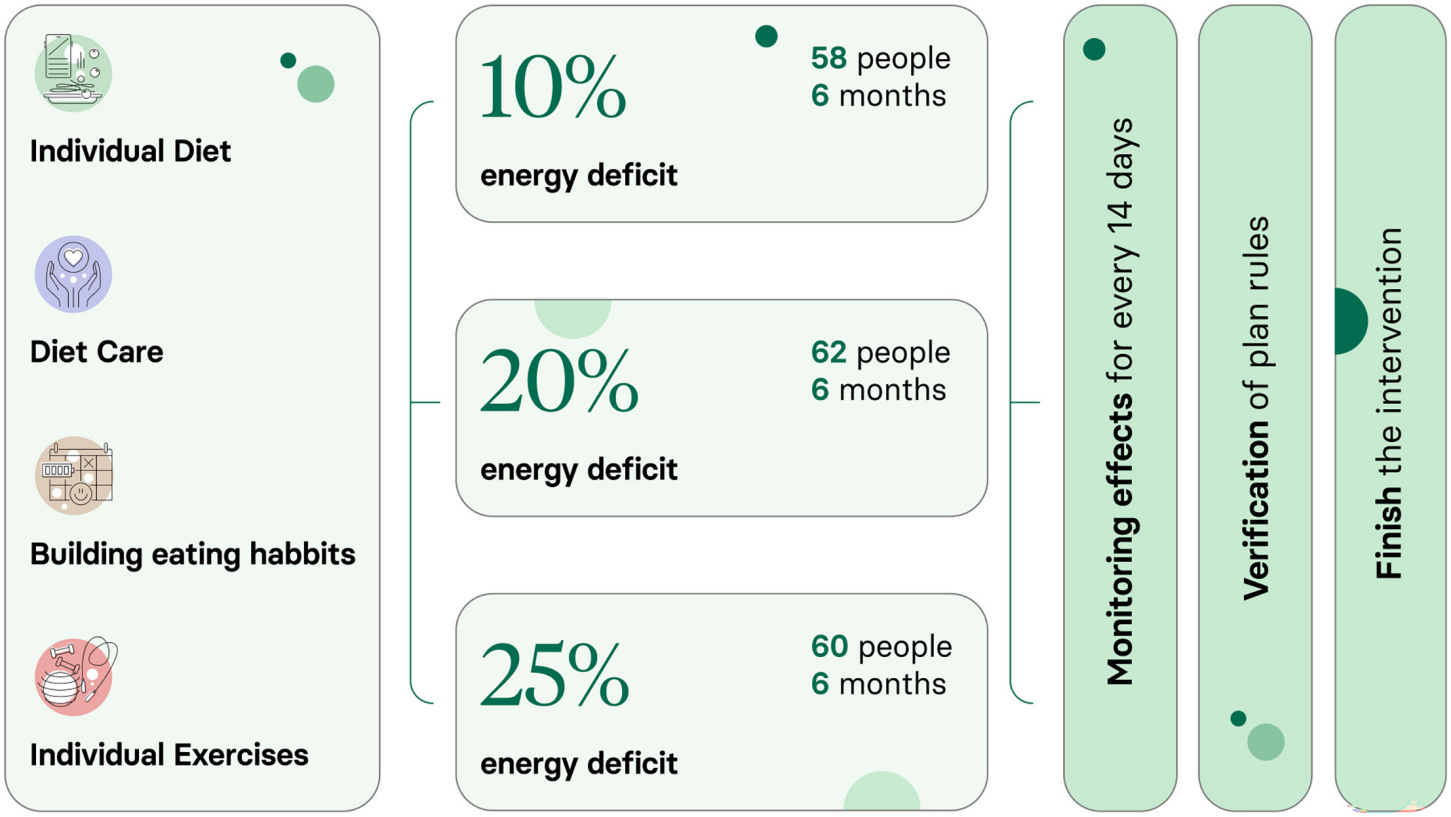
Study operation protocol.

We described the Respo method in more detail in two articles in which the method was used (Woźniak, Garbacz, et al., 2022; Woźniak, Woźniak, et al., 2022). The protocol of action is presented in Figure 1.

The dietary intervention protocol was formulated in adherence to the dietary guidelines outlined for adult populations in Poland (Jarosz, 2020). The maximum level of simple sugars in the study diets was 10% of the diet's energy content. Protein supply resulted from the level of physical activity of the patients and the strength training element in their training plan (Lewandowicz et al., 2015; Vitale & Getzin, 2019). The caloric content of the intervention diet was decreased by varying degrees—10%, 20%, or 25%—relative to individual energy needs, which were determined based on the basal metabolic rate calculated using the Harris and Benedict equation. This calculation considered the level of physical activity (PAL) as per the guidelines provided by the Institute of Food and Nutrition (Woźniak, Woźniak, et al., 2022). The assessment of physical activity levels was conducted utilizing the physical activity questionnaire (Johansson & Westerterp, 2008).

The dietary regimen was composed utilizing a program equipped with an extensive database comprising products and food items sourced from the National Institute of Public Health of the National Institute of Hygiene (PZH‐PIB) and the United States Department of Agriculture (USDA) (FoodData Central, n.d.). The surveyed people reported their compliance with the diet recommendations on an ongoing basis in a dedicated application, in which they marked the meals consumed as part of the menu and could add additional amounts of consumed food. The data obtained were used to assess compliance with dietary recommendations. The precise nutritional value of the diet is shown in Table 1.

**TABLE 1:** Nutritional value of the diet.

Variable	Characteristic
Caloric diet [% TDEE]	75 or 80 or 90
Proteins [g/kg body mass]	1.5–1.6
Fats [% of energy]	30–35
Carbs [% of energy]	45–50
Dietary fiber [g]	30–40
Saturated fatty acids [% of energy]	<5
Monosaturated fatty acids [% of energy]	14–26
Polysaturated fatty acids [% of energy]	4–6

Abbreviation: TDEE, total daily energy expenditure.

### 2.4 Outcome measurements

Body circumference, height, and weight were taken by the patients themselves following previously prepared instructions, during which the study leader (dietitian) demonstrated the correct execution of these steps. Measurements were performed by the patients every 2 weeks. Height was assessed with precision to the nearest 1 centimeter (cm), while body weight was measured with accuracy to the nearest 0.1 kilogram (kg). Body weight was measured using CE‐approved scales, which was checked at the time of qualifying patients for the study. This aspect was required to correctly qualify the subject for the intervention. All measurements were taken in underwear, on an empty stomach, in the morning. These values were used to determine BMI (body mass index). For BMI classification, the following BMI ranges were adopted according to World Health Organization (WHO) recommendations (World Health Organization, 2020). Hip and waist circumferences were measured using a tailor's centimeter to the nearest 0.1 cm.

Throughout the dietary intervention, participants maintained regular contact with both the dietitian and the trainer, providing 14 days reports including body weight and body circumference measurements. To ensure uniformity in the intervention approach, all study participants contacted a dietitian at least once or twice a day. Additionally, participants meticulously recorded their intake using a designated app, which enabled real‐time monitoring of adherence to prescribed dietary guidelines. Each participant received comprehensive instructions on the correct way to fill out the diary in the Respo application. Additionally, participants documented the frequency and volume of fluid intake as part of their dietary records in the same app.

### 2.5 Statistical analysis

The data obtained during the study were organized and structured using Excel spreadsheet tools. The spreadsheet facilitated the calculation of derived parameters, such as body mass index (BMI) and the assessment of changes in anthropometric indicators over time. The STATISTICA 13.3 PL software package was used for quantitative analysis (TIBCO Software Inc. (2017). Statistica (data analysis software system), version 13. http://statistica.io/). The significance level of *p* < .05 was assumed as the threshold for rejecting the null hypothesis in all statistical analyses.

Descriptive statistics were calculated for quantitative data, while the distributions of qualitative characteristics were estimated using multivariate (contingency) tables. The chi‐square test in combination with multivariate tables was used to assess the significance of differences in the distribution of qualitative characteristics.

Due to the rejection of the null hypothesis for most of the analyzed variables using the Shapiro–Wilk test and the expression of a significant part of the variables in ordinal scales, nonparametric tests were used. These include the Mann–Whitney *U* test (with continuity correction), Wilcoxon pairwise order test, and analysis of variance (ANOVA)–Kruskal–Wallis or Friedman's rank test, with post hoc tests when necessary.

Relationships between variables were examined using nonparametric Spearman's correlation analysis. This comprehensive analytical approach facilitated robust evaluation and interpretation of the data obtained during the study.

## 3 RESULTS

The study included 90 women and 90 men. The mean BMI without division into groups with a given energy deficit was 30.5 ± 5.0 kg/m^2^ (min 21.3–max 49.2). Among the patients, there were 86 with a BMI indicating overweight. Fifty‐four patients had class 1 obesity, while the remaining 30 patients had class 2 or greater obesity. The waist circumference of the patients averaged 101.1 ± 14.8 cm at the beginning of the intervention and the hip circumference was 106.9 ± 12.5 cm. PAL showed an activity level among patients between 1.2 and 1.9 units. The patients trained 3 times a week, with an average training time of 63 ± 10 min. The remaining general characteristics before the division into intervention groups are shown in Table 2.

**TABLE 2:** Characteristics of patients before intervention.

Variable	*n* = 180 women = 90 men = 90	
	Mean ± SD	Median (min–max)
Age [years]	34.1 ± 7	34 (21–50)
Height [m]	1.74 ± 0.1	1,74 (1.48–2.03)
Body mass [kg]	93.1 ± 20.1	91,1 (55–162.8)
BMI [kg/m^2^]	30.5 ± 5.04	29,4 (21.3–49.2)
BMR [kcal]	2048 ± 440	2003 (1210–3581)
PAL	1.43 ± 0.14	1,4 (1.2–1.9)
TDEE [kcal]	2899 ± 601	2875 (1896–4300)

Abbreviations: BMR, basal metabolic rate; MI, body mass index; PAL, physical activity level; TDEE, total daily energy expenditure.

After dividing into groups with different energy deficits, the number of patients was D10 group *n* = 58 (10% energy deficit), D20 group *n* = 62 (20% energy deficit), and D25 group *n* = 60 (25% energy deficit), respectively. At the beginning of the study, differences could be observed relative to initial body weight in each group. The D25 group was significantly different in this regard with a mean patient's weight of 98.8 ± 20.7 kg vs. 90.3 ± 22.2 kg and 90.1 ± 15.9 kg in the D10 and D20 groups (*p* = .02), respectively. The D25 group had significantly higher BMI, hip circumference, and BMR than the other two groups. The exact values are shown in Table 3.

**TABLE 3:** Characteristics of three groups of deficits before intervention.

	Deficit 10% (*n* = 58) women = 29 men = 29		Deficit 20% (*n* = 62) women = 31 mn = 31		Deficit 25% (*n* = 60) women = 30 men = 30		p**
	Mean ± SD	Median (min‐max)	Mean ± SD	Median (min‐max)	Mean ± SD	Median (min‐max)	
Age [years]	34.27 ± 3.01	34 (21–50)	34.3 ± 6.7	33.5 (21–48)	33.7 ± 7	33 (21–50)	.81
Height [m]	1.73 ± 0.1	1.74 (148–2.03)	1.73 ± 0.08	1.72 (1.54–1.93)	1.75 ± 0.1	1.78 (1.57–1.95)	.38
Body mass [kg]	90.3 ± 22.2^a^	86.7 (55–150)	90.1 ± 15.9^a^	89.5 (56–134)	98.8 ± 20.7^b^	99.6 (59.5–162.8)	.02
BMI [kg/m^2^]	29.6 ± 5.26^a^	28 (21.3–45.3)	30.1 ± 4.49^a^	29.2 (23.1–41.8)	31.9 ± 5^b^	31.8 (22.1–49.2)	.005
Waist [cm]	99.9 ± 16.3	99.5 (71–140)	98.6 ± 12.2	99.5 (70–127)	104.7 ± 15.3	104.5 (74–144)	.06
Hips [cm]	104.8 ± 127^a^	102.5 (83–149)	105 ± 11.9^a^	104.5 (74–131)	110.8 ± 12^b^	112 (82–144)	.001
BMR [kcal]	1988 ± 486^a^	1908 (1210–3301)	1982 ± 350^a^	1969 (1232–2948)	2173 ± 456^b^	2191 (1309–3581)	.02
PAL	1.44 ± 0.14	1.42 (1.2–1.8)	1.42 ± 0.14	1.43 (1.2–1.9)	1.41 ± 0.14	1.4 (1.2–1.9)	.31
TDEE [kcal]	2848 ± 655	2698 (1869–4056)	2814 ± 507	2791 (1920–3985)	3035 ± 621	3045 (1989–4300)	.13
Trainings per week (number)	3	3 (3–3)	3	3 (3–3)	3	3 (3–3)	–
Training time [min]	63.4 ± 11.6	60 (60–65)	63.6 ± 10.4	60 (60–65)	64.6 ± 10.8	60 (60–65)	.81
Training time per week [min]	190.9 ± 34	180 (175–190)	190.9 ± 31	180 (170–185)	193.5 ± 32.4	180 (175–195)	.85

^a,b^ Kruskal–Wallis test–difference between group.

**Kruskal–Wallis–one‐way analysis of variance by ranks.

After 6 months of intervention, the D10 group experienced a weight reduction of 6.6 kg (median), which translated into a relative reduction of 7.6% (median) in their body weight. Significantly greater weight reductions were recorded in the D20 group, in which participants reduced their body weight by 8.9 kg (median), which translated into a relative reduction of 9.9% (median). The D25 group achieved the largest weight reductions in both absolute and relative terms, i.e., 10.3 kg (median) and 10.3% (median), respectively. In all three groups with different magnitudes of energy deficit, the intervention weight changes in both relative and absolute terms after 6 months were statistically significant (Kruskal–Wallis test *p* = .0001). More data are presented in Table 4.

**TABLE 4:** Results obtained after 6 months in all deficit groups.

Time	Deficit 10% (*n* = 58)					Deficit 20% (*n* = 62)					Deficit 25% (*n* = 60)					
	Mean ± SD [kg]	Median (min‐max) [kg]	Change [%]	Change [kg]	p*	Mean ± SD [kg]	Median (min‐max) [kg]	Change [%]	Change [kg]	p*	Mean ± SD [kg]	Median (min‐max) [kg]	Change [%]	Change [kg]	p*	*p*
Start	90.3 ± 22.2^a^	86.7 (55–150)	–	–	–	90.1 ± 15.9^a^	89.5 (56–134)	–	–	–	98.8 ± 20.7^b^	99.6 (59.5–162.8)	–	–	–	.02
In 1 month	88.8 ± 21.6	84 (54.7–148)	−3.1^a^	−2.7	.0001	88.1 ± 15.4	88.1 (55.1–130)	−1.5^b^	−1.4	.0001	95.7 ± 19.8	96 (58–158.1)	−3.6^c^	−3.6	.0001	.0001
In 2 months	87.5 ± 21.3	82.8 (53.5–145)	−1.4^a^	−1.2		86.5 ± 15.2	86.8 (54–128.2)	−1.4^b^	−1.3		93.7 ± 19.5	94.1 (56–156.6)	−2.0^c^	−1.9		.0001
In 3 months	86.5 ± 21.2	81.1 (53–144)	−2.1^a^	−1.7		85 ± 15	85.1 (53.2–126.5)	−1.9^b^	−1.7		91.7 ± 19.1	92 (56.3–154.3)	−2.2^b^	−2.1		.0001
In 4 months	85.5 ± 20.9	80.1 (52–142)	−1.2^a^	−1.0		83.4 ± 14.7	84.1 (53–125.1)	−1.2^b^	−1.0		89.8 ± 18.8	91 (55–151)	−1.2^b^	−1.0		.0001
In 5 months	84.8 ± 21	79.7 (51–141)	−0.5^a^	−0.4		82.1 ± 14.7	82.7 (52–125.1)	−1.6^b^	−1.4		88.3 ± 18.5	88.8 (54.1–148.2)	−2.4^b^	−2.2		.0001
In 6 months	85 ± 20.8	80.1 (50.3–140.8)	+0.5^a^	+0.4		80.9 ± 14.7	80.6 (50.2–122)	−2.5^b^	−2.1		87.4 ± 18.4	89.3 (53–145.1)	+0.7^a^	+0.5		.0001
After 6 months	85 ± 20.8	80.1 (50.3–140.8)	−7.6^a^	−6.6		80.9 ± 14.7	80.6 (50.2–122)	−9.9^b^	−8.9		87.4 ± 18.4	89.3 (53–145.1)	−10.3^b^	−10.3		.0001

^a,b,c^ Difference between group ‐ Kruskal–Wallis test.

*Differences from month to month–Friedman's rank test.

**FIGURE 2:**
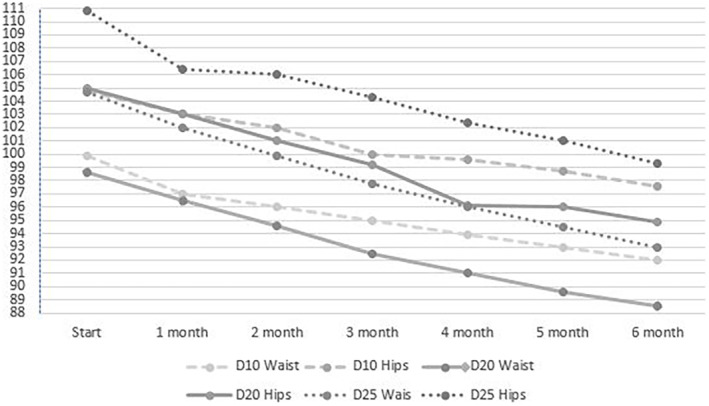
Rate of waist and hip levels over 6 months.

After 6 months of intervention, the reduction in both waist and hip circumferences at each successive month was statistically significant in all three groups (*p* = .0001). Finally, in the D10 group, the reduction in waist circumference was 7.5 cm (median) (99.5 cm vs 92 cm) and the reduction in hip circumference was 4.9 cm (median) (102.5 cm vs 97.6 cm). The D20 group recorded a reduction in waist circumference of 11 cm (median) (99.5 cm vs 88.5 cm) and a reduction in hip circumference of 9.5 cm (median) (104.5 cm vs 95 cm). The D25 group recorded a reduction in waist circumference of 11.5 cm (median) (104.5 cm vs 93 cm) and a reduction in hip circumference of 13 cm (median) (112 cm vs 99 cm). Both waist circumference reduction and hip circumference reduction after 6 months were statistically significant in each group (*p* = .0001, Kruskal–Wallis test). Post hoc analysis showed a difference in waist circumference and hip circumference reduction between the D10 vs D20 and D25 groups (*p* = .00001). In the D10 group, they were significantly smaller than in the other two groups. In the D20 and D25 groups, the differences in the decrease in body circumferences were not statistically significant (*p* = .052). The rate of decrease in waist and hip circumferences over the 6 months of the intervention is shown in Figure 2.

After the intervention, further cooperation aimed at weight reduction was declared by 93 patients, which is 51.7% of the patients (Yes group), while 87 patients declared no further desire to reduce weight in accordance with the program (No group). Patients in the Yes group had higher initial and final body weights (median before 97 kg, after 86.2 kg) compared to the No group (median before 86.5 kg, after 78.2 kg). In addition, the Yes group achieved a higher weight loss for the entire 6 months of the intervention, i.e., 10.9% (median), and in the final month of the intervention, i.e., 1.4% (median), compared to the No group, which recorded a weight loss of 6.6% for 6 months (median) and 0.8% in the final month (median), respectively. Initial as well as final BMI was also a differentiating factor between the groups. The Yes group had an initial BMI of 31.9 kg/m^2^ (median) and a final BMI of 28.4 kg/m^2^ (median), while the No group had an initial BMI of 28.4 kg/m^2^ (median) and a final BMI of 26.1 kg/m^2^ (median). All differences between the groups were statistically significant (Mann–Whitney *U* test). More results describing the two groups are presented in Table 5.

**TABLE 5:** Characteristics of two groups after intervention: The group continuing dietary cooperation (Yes), the group not continuing dietary cooperation (No).

	Group continuing dietary cooperation (yes) women = 44 men = 49		Group not continuing dietary cooperation (No) women = 46 men = 41		Difference between group*
	Mean ± SD	Median (min‐max)	Mean ± SD	Median (min‐max)	*p*
Age [years]	34.3 ± 6.9	34 (21–50)	33.9 ± 7.1	34 (21–50)	.71
Start Body mass [kg]	99.2 ± 20.3	97 (62–162.8)	86.6 ± 17.7	86.5 (55–150)	.0001
Finish Body mass [kg]	88.5 ± 18.8	86.2 (55.3–145.1)	79.9 ± 16.4	78.2 (50.2–140.8)	.0001
Start BMI [kg/m^2^]	32.4 ± 5.41	31.9 (21.3–49.2)	28.6 ± 3.7	28.4 (22.1–38)	.0001
Finish BMI [kg/m^2^]	28.8 ± 5.04	28.4 (19.3–43.5)	26.4 ± 4.7	26.1 (21.6–35.1)	.0001
Start Waist [cm]	105.2 ± 15.1	105 (70–144)	96.7 ± 13.2	98 (71–130)	.0001
Finish Waist [cm]	93.8 ± 14.2	95 (63–134)	88.4 ± 11.9	88 (65–120)	.0001
Start Hips [cm]	111 ± 13.4	110 (74–149)	102.4 ± 9.5	102 (82–133)	.0001
Finish Hips [cm]	99.9 ± 11.9	99 (66–135)	94.4 ± 8.9	94 (77–119)	.0001
Change Body mass [%]	−10.8 ± 2.0	−10.9 (5.6–14.9)	−7.6 ± 2.1	−6.6 (0.4–12.6)	.0001
Change Body mass in 6 months [%]	−1.3 ± 1.5	−1.4 (1.0–2.5)	−0.2 ± 0.2	−0.8 (0.09–0.4)	.0001

*Mann–Whitney *U* test.

## 4 DISCUSSION

The results observed show that an online comprehensive weight loss intervention targeting people with excessive body weight can be an effective tool in successfully reducing body weight, waist circumference, and hip circumference. The Respo method combines four elements related to effective weight reduction, i.e., personalized diet with energy deficit, individually tailored physical activity, constant contact with a dietitian through an online application, and support for building healthy eating habits (Grzybek et al., 2006; Haas et al., 2019). Regular feedback and motivating patients every day to change their lifestyle is the new standard of dietary care aimed at reducing excess body weight (Wing et al., 2006). In addition, the ability to monitor one's physical activity and easily report on workout performance stimulates motivation to raise one's activity factor (Thomas et al., 2009).

A strong feature of our study is the equal number of men as well as women (their share was 50% each), since in studies targeting weight loss, women outnumber men in the vast majority of publications. In the study by (Pagoto et al., 2012), men accounted for 27% of the patients, while in another study similar to ours, the number of men was only 20% (NWCR facts [webpage on the internet], 2014). An explanation for this kind of overrepresentation of women could be social issues related to society's pressure for a slim female figure or less cultural pressure on men to take care of their health. Our results indicate that significant reductions in body weight were noted in all three groups with different sizes of energy deficit. However, even a nominally significant change in body weight can sometimes prove insufficient for patients expecting individually satisfying results from dietary cooperation. Often, too little results, in the patient's mind, can demotivate those wanting to significantly reduce their body weight, and this translates into a willingness to forgo intervention. Such a trend was observed in our study, in which, of the 80 patients who dropped out of the study, as many as 67.5% (54 people) were assigned to the group with a 10% energy deficit (representing 93.1% of people in this group), and weight loss did not exceed 2.4 kg in this subgroup. Ultimately, patients in the D10 group reduced their body weight by 6.6 kg or 7.6% (median), which was a significant result, but at the lower end of the recommended weight loss of 1%–2% per month, which, in the case of a 6 months intervention, would be associated with a total weight loss of about 6%–11.5% relative to initial body weight. And while some recommendations express the recommended weight loss in kilograms, we believe that defining this parameter in percentages is much more accurate, as it takes into account baseline body weight as a baseline factor (Olszanecka‐Glinianowicz et al., 2023). The recommended ranges for weight loss after 6 months included the D20 and D25 groups. Patients in these groups reduced their body weight by 9.9% and 10.3% (median), respectively. In these two groups, only 26 patients (representing 21.3% of those in both groups) chose to discontinue the protocol, which may suggest a positive effect of the intervention's effects on the willingness to remain in the program (Hartmann‐Boyce et al., 2014; Hayes & Hofmann, 2017; Jacob & Isaac, 2012; Wadden et al., 2014). Ultimately, we consider that an energy deficit of 20% is sufficient and optimal to achieve effective weight loss results over a 6‐month period. The 25% deficit had better results, but the results were not greater in terms of clinical significance, and too high an energy deficit may be associated with lower satisfaction among dieters, lower levels of motivation to follow a more rigorous diet, or an increased risk of snacking while dieting. Therefore, a level of 20% energy deficit seems to be the optimal solution taking into account the risks and benefits of the intervention.

The D25 group, compared to the D20 group, had a higher baseline body weight and a higher BMI. The difference between the two groups was 8.7 kg (median) and 1.8 BMI kg/m^2^ (median), respectively. However, their activity level and the amount and length of training were the same, which made it possible to objectively compare these groups with each other considering the percentage weight loss rate. As expected, the difference in weight loss between the groups was not large. However, a surprise is the relatively high weight loss in the D10 group, which had a low energy deficit compared to the D20 and D25 groups. Possible explanations for this could be the patients' undereating in the D10 group despite careful monitoring, or the patients' eating between meals in the D20 and D25 groups, but this was not recorded by the study participants in their food diaries. It is worth bearing in mind this kind of risk of deviation from the norm when the study is conducted without full control of caloric intake as in closed center studies (Kalm & Semba, 2005).

The weight loss in all groups should be considered significant and clinically meaningful. In a comparable study in which the researchers applied a caloric deficit of the order of 500 kcal in a group of 35 patients with a mean BMI of 35.3 ± 5.7 kg/m^2^ and body weight of 102.1 ± 20 kg, the weight loss after 6 months of intervention was 11.3% (Luley et al., 2011). Unfortunately, the researchers did not estimate the total metabolism of the patients by which it is not possible to verify whether the energy deficit was expressed as a percentage. In our study, the level of absolute deficit after conversion from percentages was for the groups, respectively: D10–270 kcal (median), D20–558 kcal (median), and D25–761 kcal (median). In another study lasting 6 months using a 1500 kcal diet for women and 1800 kcal diet for men, the patients reduced their body weight by 7% using a low glycemic index diet and by 3.2% using a conventional diet (Foster et al., 2003). Similar results in weight loss were reported by Samaha et al. (Samaha et al., 2003) in whose study patients reduced their body weight by 5.8%.

Given the well‐studied yo‐yo effect in weight‐loss patients (Di Germanio et al., 2018), we focused on finding out differences between the group that opted for further dietary cooperation (Yes group) and those who did not want further help (No group) that might influence minimizing this effect. After analysis, we believe that the key factors influencing the desire for longer dietary cooperation than 6 months are higher baseline body weight and/or higher BMI. Probably due to the desire to reduce one's weight by a higher number of kilograms, which translates into a longer collaboration. The rate of weight loss is also not insignificant for the patient to want to continue the dietary cooperation. After analyzing the results of our study, we believe that a continuous and high weight loss of at least an average of 1.5% of body weight per month has a positive effect on the patient's motivation and desire for further weight reduction.

### 4.1 Limitations of the study

The study encountered several limitations. Primarily, due to the nature of the online intervention, it was not possible to absolutely confirm participants' adherence to the protocol with 100% certainty. Although efforts were made to monitor program implementation throughout the intervention period, full assurance of compliance remained elusive. Furthermore, the duration of the study was limited to 6 months, which made it impossible to assess participants' ability to maintain body weight after the intervention. The lack of assessment of weight maintenance after the intervention represents a notable gap in the study results. During the study, changes in eating habits among the respondents were not monitored.

Additionally, technological limitations made it difficult to measure energy expenditure during physical activity and throughout the day, which could potentially affect the observed results. The inability to quantify energy expenditure represents a significant limitation to a comprehensive understanding of the mechanisms underlying the observed results. Furthermore, the study did not allow for a direct comparison between the mobile‐based lifestyle intervention group and the offline intervention group, making it impossible to assess the relative effectiveness of online and offline interventions. Such a comparison could provide valuable information about the effectiveness and applicability of different intervention methods.

Taken together, these limitations highlight the need for careful interpretation of study results and point to opportunities for future research to address methodological gaps and increase the robustness of intervention strategies in similar contexts.

## 5 CONCLUSIONS

In conclusion, taking all factors into account, it seems that an energy deficit of 20%–25% is most appropriate in terms of weight reduction lasting 6 months. The level of weight reduction is then clinically relevant and motivating for patients paying attention to the effects of the diet. In turn, the key factors positively influencing patients' willingness to extend the collaboration beyond 6 months are baseline BMI and the amount of weight reduction during the intervention. The results of our study indicate that an online dietary intervention, along with regular physical activity and ongoing patient support, aimed at reducing excess body weight is an effective method of collaboration with patient with obesity.

## AUTHOR CONTRIBUTIONS

Data curation, J.W. and K.W.; formal analysis, J.W., M.W., and D.W.; methodology, J.W. and D.W.; project administration, J.W.; supervision, D.W. and M.W.; writing—original draft, J.W., K.P., and K.W.; writing—review and editing, D.W. and M.W. All authors have read and agreed to the published version of the manuscript.

## FUNDING INFORMATION

The study was not funded.

## CONFLICT OF INTEREST STATEMENT

The authors declare no conflict of interest.

## INSTITUTIONAL REVIEW BOARD STATEMENT

The local Ethics and Scientific Research on Humans Commission of Faculty of Human Nutrition and Consumer Sciences—SGGW (Warsaw University of Life Sciences) approved the research project (approval number: 35/2021).

## Data Availability

Data available on request.
